# Repellent Activities of Essential Oils of Some Plants Used Traditionally to Control the Brown Ear Tick, *Rhipicephalus appendiculatus*

**DOI:** 10.1155/2014/434506

**Published:** 2014-02-19

**Authors:** Wycliffe Wanzala, Ahmed Hassanali, Wolfgang Richard Mukabana, Willem Takken

**Affiliations:** ^1^Department of Biological Sciences, School of Pure and Applied Sciences, South Eastern Kenya University, P.O. Box 170-90200, Kitui, Kenya; ^2^Behavioural and Chemical Ecology Department, International Centre of Insect Physiology and Ecology, African Insect Science for Food and Health, P.O. Box 30772-00100-GPO, Nairobi, Kenya; ^3^Chemistry Department, School of Pure and Applied Sciences, Kenyatta University, P.O. Box 43844-00100-GPO, Nairobi, Kenya; ^4^School of Biological Sciences, University of Nairobi, P.O. Box 30197-00100-GPO, Nairobi, Kenya; ^5^Laboratory of Entomology, Wageningen University and Research Centre, P.O. Box 8031, 6700 EH Wageningen, The Netherlands

## Abstract

Essential oils of eight plants, selected after an ethnobotanical survey conducted in Bukusu community in Bungoma County, western Kenya (*Tagetes minuta, Tithonia diversifolia, Juniperus procera, Solanecio mannii, Senna didymobotrya, Lantana camara, Securidaca longepedunculata*, and *Hoslundia opposita*), were initially screened (at two doses) for their repellence against brown ear tick, *Rhipicephalus appendiculatus*, using a dual-choice climbing assay. The oils of *T. minuta* and *T. diversifolia* were then selected for more detailed study. Dose-response evaluations of these oils showed that *T. minuta* oil was more repellent (RD_50_ = 0.0021 mg) than that of *T. diversifolia* (RD_50_ = 0.263 mg). Gas chromatography-linked mass spectrometric (GC-MS) analyses showed different compositions of the two oils. *T. minuta* oil is comprised mainly of *cis*-ocimene (43.78%), dihydrotagetone (16.71%), piperitenone (10.15%), *trans*-tagetone (8.67%), 3,9-epoxy-p-mentha-1,8(10)diene (6.47%), **β**-ocimene (3.25%), and *cis*-tagetone (1.95%), whereas *T. diversifolia* oil is comprised mainly of **α**-pinene (63.64%), **β**-pinene (15.00%), isocaryophyllene (7.62%), nerolidol (3.70%), 1-tridecanol (1.75%), limonene (1.52%), and sabinene (1.00%). The results provide scientific rationale for traditional use of raw products of these plants in controlling livestock ticks by the Bukusu community and lay down some groundwork for exploiting partially refined products such as essential oils of these plants in protecting cattle against infestations with *R. appendiculatus*.

## 1. Introduction

In sub-Saharan Africa, East Coast fever (ECF), caused by *Theileria parva parva,* Theiler, 1904, and transmitted by the brown ear tick, *Rhipicephalus appendiculatus,* Neumann, 1901, is one of the major constraints to the development of the livestock industry [1, 2]. Of the estimated 12.7 million head of cattle (both indigenous and exotic), 76% are at risk to ECF [3]. The disease is associated with up to 10% mortality in zebu calves in ECF endemic areas and can cause up to 100% mortality in susceptible exotic and indigenous breeds [3, 4].

Prevention, control, and management of both vector and pathogen have continued to rely heavily on the application of synthetic chemical acaricides on the host since their introduction in 1902 in sub-Saharan Africa [5]. However, this has proved to be costly and unsustainable in a number of ways [1]. The acaricides can eliminate ticks from the host, but they do not prevent continued reinfestation from the source environment, where ticks spend 90% of their life. For effective management of harmful ticks, an integrated combination of tactics may need to be put in place that controls ticks on individual hosts as well as in the host environment in order to prevent host reinfestation during grazing. One possible strategy would be to use tick repellents on the host and tick-repellent plants in the pasture (host environment), combined with plants that are attractive to ticks such as *Acalypha fruticosa *Forssk. var. villosa Hutch (Family: Euphorbiaceae) surrounding the pasture land so as to develop a “push-pull” tick manipulation system [6, 7]. Although the proposed strategy appears complex, it may be possible to achieve in zero/semizero grazing, small-scale free-range, and tethering livestock farming systems. In others, such as pastoralism and large-scale livestock farming systems, the deployment of well-formulated repellents dispensed from controlled-release dispensers may be more practical.

N,N-diethyl-3-methylbenzamide (DEET) is still considered the best available product, repelling a wide variety of insects, ticks, and mites [8]. Though DEET is not expected to bioaccumulate, the amounts present in the environment have been shown to be toxic to some species of zooplankton and fish [9, 10]. In humans, the repellent may cause insomnia, mood disturbances, impaired cognitive functions, seizures, toxic encephalopathy, and allergic reactions [11–13]. This has led to a search for alternative eco-friendly and effective repellents.

The potential of some local plants and plant products to repel ticks from grazing areas and host animals, respectively, has been demonstrated previously [14–18]. *Melinis minutiflora* (molasses grass), a tropical grass already in use as livestock fodder [19, 20], covers crop and mulch [21] and for thatching houses [22], it has been shown to be toxic [15] and repellent to ticks [14, 23, 24] as well as insects and snakes [21]. One study demonstrating potential of molasses grass to control *Amblyomma variegatum* and *R. appendiculatus*, vectors of the livestock diseases heartwater (cowdriosis) and ECF, respectively, has been reported [24]. In addition, several other Kenyan local shrubs, including *Cleome hirta* and *Gynandropsis gynandra*, have demonstrated potential as tick-repellent pasture plants [25–27].

A number of studies have shown that plant-based repellents can be comparable to DEET or even better [28–32]. One commercial repellent product is the Flea and Tick Granular Repellent, which is made from essential oils of cedar, cinnamon, mint, and lemon grass; it has a pleasant odour and can be safely used outdoors for flea and tick control [33]. Essential oils of a number of other plants have been shown to be repellent to ticks. These include *Commiphora erythraea* and *C. myrrh* [34], *Cleome monophylla* [35], *Ocimum suave* [36], *Cleome hirta* [27], and *G. gynandra* [37].

Use of tick-repellent plants in pasture lands or essential oils on hosts and their integration with other off-host or on-host tick control measures could be practical and provide economic ways of controlling not only livestock ticks but also arthropod vectors [38–40]. In our previous survey of livestock tick control ethnopractices among Bukusu community in Bungoma district, western Kenya, we found widespread use of ethnobotanicals derived from local/native plants to control tick infestations on cattle [41]. Blends of botanicals from one or more plants are used either as on-host suspensions or burnt and smoke used to fumigate cattle. Our follow up objective has been to assess the repellence of essential oils of some of these plants against *R. appendiculatus* adults in the laboratory, to characterize the chemical constituent profiles of the more repellent ones, and then to initiate both off- and on-host evaluation of their efficacy in controlling the ticks in the field. In the present paper, we report the results obtained from repellence assays of essential oils of 8 plants against *R. appendiculatus* adults and results of a more detailed study of two selected plants, *Tagetes minuta* L. and *Tithonia diversifolia* (Hemsl.) A. Gray.

## 2. Materials and Methods

### 2.1. Selection of Eight Plant Species

An ethnobotanical survey was previously conducted in the Bukusu community in Bungoma County, western Kenya, along the southern slopes and foothills of Mount Elgon at altitudes ranging from about 1,300 m in the south to about 3,500 m in the north [41]. The County is located between latitude 0°25′S and 0°53′N and longitude 34°21′W and 35°04′E. Specimens of ~157 plant species, which were documented to have varied effects on livestock ticks [41], were collected for taxonomic examination at the herbarium of the School of Biological Sciences, University of Nairobi, Kenya. The potential efficacy of each plant species in protecting cattle against tick infestations was assessed following a four-level protocol proposed by Heinrich and coworkers [42], and eight plant species were selected for initial laboratory screening [41, 43]. Voucher specimens of these plants were deposited at the University of Nairobi Herbarium, and comprised of *Tagetes minuta* L. (029-BGM-Mwi/2002), *Tithonia diversifolia* (Hemsl.) A. Gray (015-BGM-Muf/2002), *Juniperus procera* Endl. (134-BGM-Elg/2002), *Solanecio manii* (Hook. f.) C. Jeffrey. (106-BGM-Mwi/2002), *Senna didymobotrya* (Fresen.) H. S. Irwin and Barneby (132-BGM-Web/2002),* Lantana camara* L. (043-BGM-Mwi/2002), *Securidaca longepedunculata* Fres. (018-BGM-Mec/2002), and *Hoslundia opposita *Vahl. (133-BGM-Bul/2002).

### 2.2. Experimental Ticks

The ticks used (the brown ear tick, *Rhipicephalus appendiculatus* Neumann, 1901) were obtained from the colonies at the International Livestock Research Institute (ILRI) and bred in the insectary at ICIPE, Nairobi, Kenya. Rearing conditions and management of ticks were as described previously [44, 45]. All the experiments were conducted using the newly emerged adult ticks of mixed sexes.

### 2.3. Isolation of Essential Oils

The aerial parts of each of the eight plants were collected from the southern slopes and foothills of Mount Elgon in western Kenya during the month of August and allowed to dry in a well-ventilated room for 1-2 weeks. Each plant material was cut into small pieces and about 1 kg was hydrodistilled using a Clevenger-type apparatus for 8 h [46]. Essential oil of each plant was collected in 2 mL vials and stored at −20°C in a freezer until required for bioassays or analyses.

### 2.4. Dual-Choice Repellence Assays

A dual-choice tick repellence climbing assay [47] that exploits the behaviour of *R. appendiculatus* to climb up grass stems to await potential hosts passing by [48, 49] was used. The repellence of essential oils of the eight plants against *R. appendiculatus* was first compared at 0.1 mg and 50 mg doses. The most repellent oils (that of *T. minuta* and that of *T. diversifolia*) were then selected for more detailed study. These plants are also highly ranked by livestock holders of Bukusu community in livestock tick prevention and control [41]. The oils of the plants were diluted serially with dichloromethane (GC grade) to provide 0.5 mg to 0.00005 mg/10 *μ*L of solutions. An aliquot of 20 *μ*L of each dose was applied to filter paper strip on the glass tubes, with an equivalent volume of dichloromethane added to the control filter paper strip. The-set up was allowed to equilibrate for 30 min before five adult* R. appendiculatus* of mixed age and sex were released on the base of the climbing set-up assay [47]. Observations were made over a 1-hour period, and the number of ticks above the filter paper strip on the control glass tube (Nc) and on the glass tube with test materials (Nt) was recorded at 15, 30, 45, and 60 min. Twenty replicates for each dose were carried out, each time with fresh, naïve adult ticks. Initial comparison of the responses of ticks in the set-up with and without residual dichloromethane on both sides showed no bias for either side and no effects of the residual solvent on the adult ticks. The repellency of each dose was calculated using the formula: (number of ticks in control arm − number of ticks in treated arm/total responding ticks) × 100. Dose-response data were subjected to probit analysis using the % repellencies from the replicated experiments [47].

### 2.5. Determination of the Composition of *T. minuta *and *T. diversifolia* Essential Oils

GC-MS analyses of the two oils were performed with a VG Masslab 12-250 quadruple gas chromatography-mass spectrometer. Chromatographic separations were achieved using a fused silica capillary column (Hewlett Packard, 50 m × 0.32 mm ID) coated with Carbowax 20 M (0.3 *μ*m film thickness) with helium as the carrier gas. All the GC-MS analyses were made in the splitless mode with helium as the carrier gas. The oven temperature was programmed from 60°C for 7 min, to 120°C at 5°C per min, then to 180°C at 10°C per min, and finally to 220°C at 20°C per min, where it was maintained for l0 min. Constituents of the essential oils were identified by analysis of their mass spectra, direct comparison of these with those in the Wiley NBS and NIST databases, and coinjections with authentic standards (from Sigma Chemical Company, Poole, UK and Aldrich Chemical Company, Gillingham, UK) on a Hewlett Packard HP 5890A Gas Chromatograph equipped with a flame ionization detector (at 230°C). A fused silica capillary column (Hewlett Packard, 50 m × 0.22 mm × 0.33 mm CD) coated with methyl silicon (0.3 *μ*m film thickness) was used with nitrogen as the carrier gas. All GC analyses were performed in the splitless mode with the injector temperature at 270°C and oven temperature programme similar to that in GC-MS analyses.

### 2.6. Data Analysis

Dose-response data were subjected to simple regression and probit analysis using the percent repellency values obtained from replicated experiments and a regression model developed based on

(1) $$\begin{array}{c} \;\;\;\;\;\;\text{Probit}\left[\Pi \left(\text{dose}1\right)\right]=\beta_{0}+\beta_{1}x\;+\in , \end{array}$$

where *β*_0_ is the coefficient of the model representing *y*-intercept, *β*_1_ is the coefficient of the model representing dose1, *x* is the various concentrations of essential oils, dose1 is the Log_10_ (dose), ∈ is the error term (residual term) representing the difference between the actual observed value and that predicted by the model (the predictor (regressor) variable, *x* is the dose of the essential oil), and Π is the repellency probability.

Student-Newman-Keuls *H* test was used to compare the mean values of repellency obtained for various doses of the repellent effects [50]. Percent repellency values were transformed into probabilities, while essential oil doses were transformed into logarithms to base 10 and lines for regression models fitted using R software for Microsoft windows. These models were used to estimate repellent effects of the two essential oils at RD_50_ and RD_75_ [8, 51].

## 3. Results

### 3.1. Screening of the Essential Oils Isolated from the Selected Eight Plant Species

**Figure 1:**
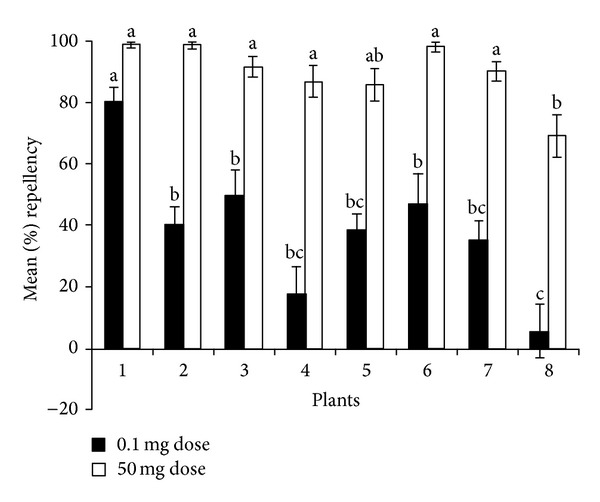
The repellent effect of essential oils of eight plants at doses of 0.1 mg and 50 mg (neat oil) against newly emerged *Rhipicephalus appendiculatus* adults. Plant species 1 is* Tagetes minuta*, 2 is *Tithonia diversifolia*, 3 is *Hoslundia opposita*, 4 is *Solanecio mannii*, 5 is* Lantana camara*, 6 is *Juniperus procera*, 7 is *Senna didymobotrya* and 8 is *Securidaca longepedunculata*. For a given repellent dose, means capped by the same alphabetical letters are not significantly different at *P* < 0.0001 (Student-Newman-Keuls *H* test).

The results of repellency tests following the screening of the essential oils isolated from the eight plants (*T. minuta*,* T. diversifolia*,* J. procera*, *S. mannii, S. didymobotrya*,* L. camara*, *S. longepedunculata,* and *H. opposita*) at 0.1 mg and 50 mg doses are shown in Figure 1. Some variation was found in the repellent effect of the essential oils at the two doses with that of* S. longepedunculata* showing the least repellent effect at both doses and that of *T. minuta* showing the highest repellent effect at the lower dose (80.1 ± 4.7%). The essential oil of *T. minuta* and one of the other six plants (*T. diversifolia*) were therefore selected for more detailed bioassay.

### 3.2. Dose-Response Repellency of the Essential Oils of *T. minuta* and *T. diversifolia*

**Figure 2:**
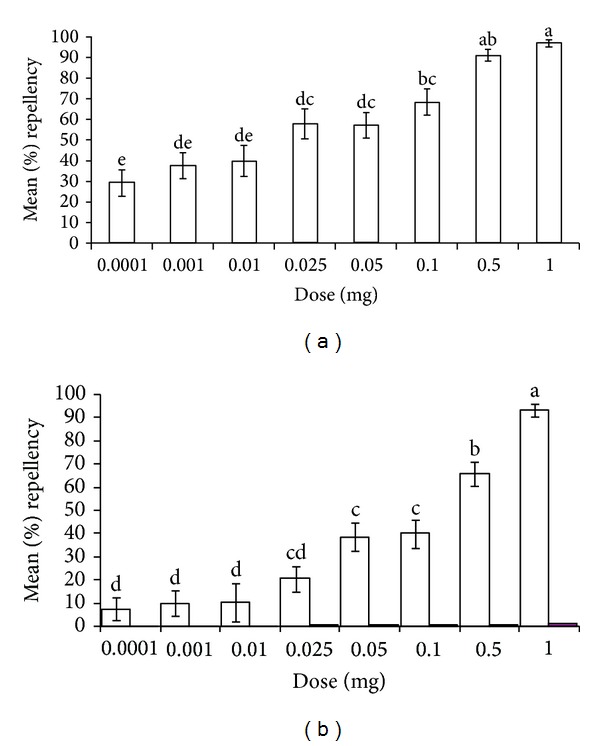
Mean repellency percentage of different doses of *Tagetes minuta* (a) and *Tithonia diversifolia* (b) essential oils against newly emerged adults,* Rhipicephalus appendiculatus,* in a dual-choice assay. Means with the same alphabetical letters are not significantly different at *P* < 0.0001 (Student-Newman-Keuls *H* test).

The repellence of the two essential oils at different doses is shown in Figures 2(a) and 2(b). The essential oil of *T. minuta* was found to be significantly more repellent than that of *T. diversifolia* at all corresponding doses (*P* < 0.05). In both the essential oils of *T. minuta *and *T. diversifolia*, there was significant correlation between repellence and dose (Pearson Correlation, *α* = 0.01). Model development of the bioassay data of the two essential oils allowed estimation of RD_50_ and RD_75_ (Table 2). Previous work at ICIPE, Nairobi, Kenya, tested various DEET doses under the same laboratory conditions as described previously [27, 35, 37] and determined their percent repellence against *R. appendiculatus* (Table 3), with which we compared the current dose-response repellencies caused by the essential oils of *T. minuta* and *T. diversifolia*.

### 3.3. Major Chemical Components of the Essential Oils of *Tagetes minuta* and *Tithonia diversifolia*

Gas chromatography (GC) in combination with gas chromatography/mass spectrometry (GC-MS) separated the chemical components in the mixtures of the essential oils of *T. minuta* and *T. diversifolia* plants, and the major representative GC/GC-MS profiles are shown in Tables 1(a) and 1(b), respectively. The major chemical components of *T. minuta *essential oil were *cis-*ocimene (43.78%), dihydrotagetone (16.71%), piperitenone (10.15%), *trans*-tagetone (8.67%), 3.9-epoxy-p-mentha-1.8(10)diene (6.47%), *β*-ocimene (3.25%), *cis*-tagetone (1.95%), and *β*-caryophyllene (0.84%). Those chemical components of the essential oil of *T. diversifolia* were mainly **α**-pinene (63.64%), *β*-pinene (15.00%), isocaryophyllene (7.62%), nerolidol (3.70%), 1-tridecanol (1.75%), limonene (1.52%), and sabinene (1.00%).

## 4. Discussion 

In a previous study, we undertook a survey of indigenous knowledge of the Bukusu community of western Kenya on livestock ticks, the risks they pose and ethnopractices associated with their management [41]. The study showed that the Bukusu community has accumulated rich ethnoveterinary knowledge and practices and that on-host use of ethnobotanical suspensions and fumigation of host animals with volatiles from burning plant products (prepared from one or more of ~157 plants) constitute important methods of controlling the ticks. In the present study, repellence of essential oils associated with some of the plants was used to assess one possible mode of action of the plant products on ticks. Eight of these plants were selected for screening against the adults of the brown ear tick following a four-level assessment protocol (based on additional ethnobotanical information on similar use of the plant elsewhere, reported phytochemical profile of the plant or related species, and any documented bioactivity data of the plant extracts or their phytochemicals) proposed by Heinrich and coworkers [42]. The essential oils of these plants showed some variation in repellence against newly emerged *R. appendiculatus* adult ticks. This was particularly apparent at the lower dose (0.1 mg), with the essential oil of *T. minuta* showing the highest repellence and that of *S. longepedunculata* showing the least repellent effect.

Comprehensive repellence studies with *T. minuta* and *T. diversifolia *oils at eight doses confirmed the higher repellence of the former against *R. appendiculatus.* Interestingly, its repellent effect is comparable to that of commonly used repellent DEET at 0.1 mg dose (with essential oil of *T. minuta* producing a repellent effect of 80.1 ± 4.9% compared with that of DEED, 84.0 ± 3.9%). Additionally, the results obtained by this study are compared favourably with the results reported by Nchu and coworkers [52] for the essential oil of *T. minuta* plants growing in Pretoria, South Africa, against *Hyalomma rufipes* Koch. *H. rufipe*s is also widely distributed in Africa and can transmit disease-causing viral and protozoan (e.g., Crimean-Congo haemorrhagic fever and Babesia, resp.) pathogens to livestock and humans alike [53, 54]. Although the *T. minuta* oil obtained in the present study shares a series of major constituents (e.g., *cis*-ocimene, dihydrotagetone, piperitenone, tagetone, and *β*-ocimene) with that isolated in Pretoria, South Africa [52], there are also some chemotypic differences between the two, reflected in different proportions of these compounds and the presence of some constituents in one chemotype (e.g., 3-methyl-2-(2-methyl-2-butenyl)-furan in *T. minuta *oil collected in Pretoria, South Africa) that were not detected in the other. Which constituents contribute to the repellence of the essential oil of *T. minuta* against the two tick species, respectively, must await detailed subtractive assays [55] with synthetic blends of the major constituents of the two essential oils with each component missing at a time.

Nchu and coworkers [52] also found that *T. minuta* oil significantly delayed moulting of *H. rufipes *engorged nymphs. In another study reported by Krishna and coworkers [56], most eggs of the coleopteran beetle, *Tribolium castaneum*, exposed to the vapours of essential oil of a specific genotype of *T*. *minuta* failed to hatch. Both these effects have been attributed to tagetone, one of the major constituents of *T. minuta* previously shown to have growth disrupting bioactivities on arthropods [57]. Since *trans*-tagetone is a prominent constituent of *T. minuta* essential oil of Bungoma chemotype (8.7%, compared with 1.6% in the Pretoria chemotype), it will be interesting to see if similar repellent effects are also observed with nymphal *R. appendiculatus*.

In this study, essential oil of *T. diversifolia* growing in Bumgoma was found to be less repellent than that of *T. minuta*. This is reflected in its very different terpenoid profile. However, the plant is highly valued for its tick control property by the Bukusu community in Bungoma [41]. Interestingly, it is also used by the Kikuyu community in central Kenya to control livestock ticks [58] and by the Luyha and Luo communities in western Kenya to control insect pests in arable farming systems [59, 60]. This suggests other possible modes of action of *T. diversifolia *phytochemical profile. Follow-up studies on other potential modes of action of the essential oil and nonvolatile constituents of the plant may help to shed light on this question.

## 5. Conclusion 

In conclusion, the present study provides some scientific rationale for the incorporation of some botanicals in Bukusu ethnopractices in western Kenya to control tick infestations on cattle. The study also lays down some groundwork for follow-up studies on other possible effects of the phytochemicals of the plants studied and for exploiting partially refined products such as essential oils in protecting cattle against infestations by *R. appendiculatus* and other tick species.

## Figures and Tables

**Table:** (a)

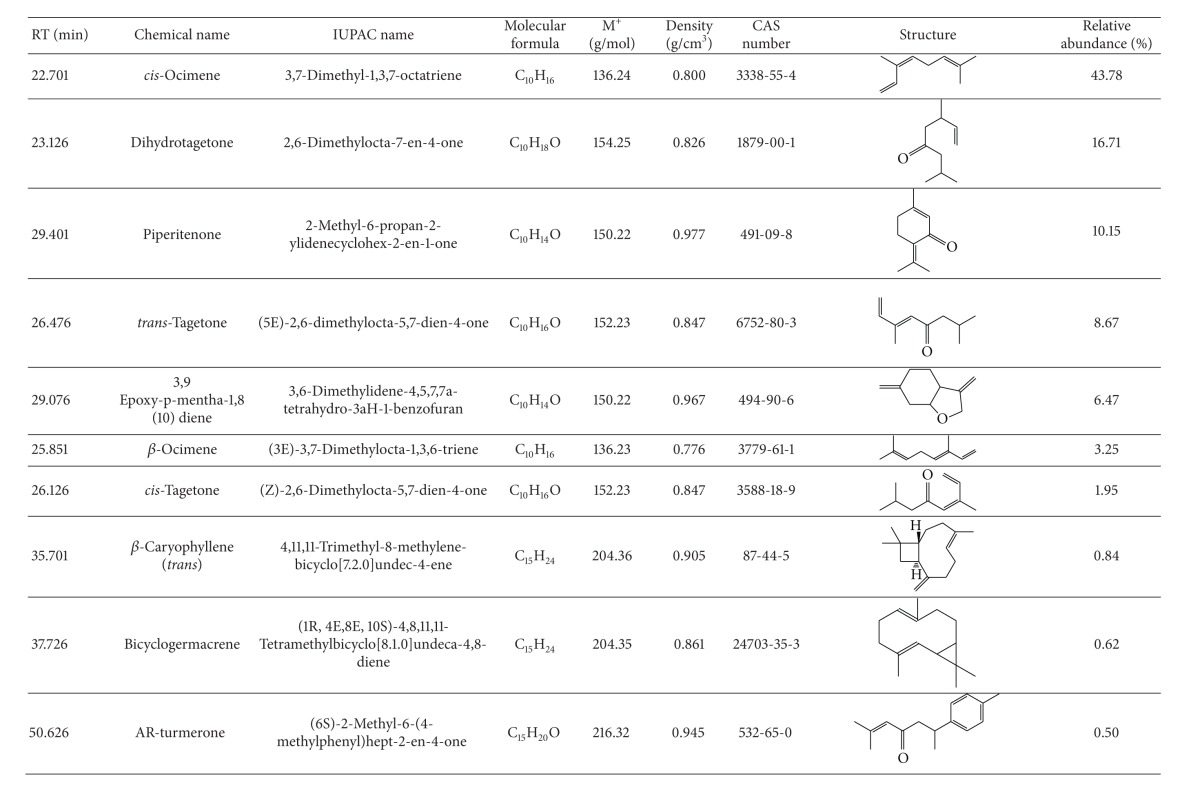

**Table:** (b)

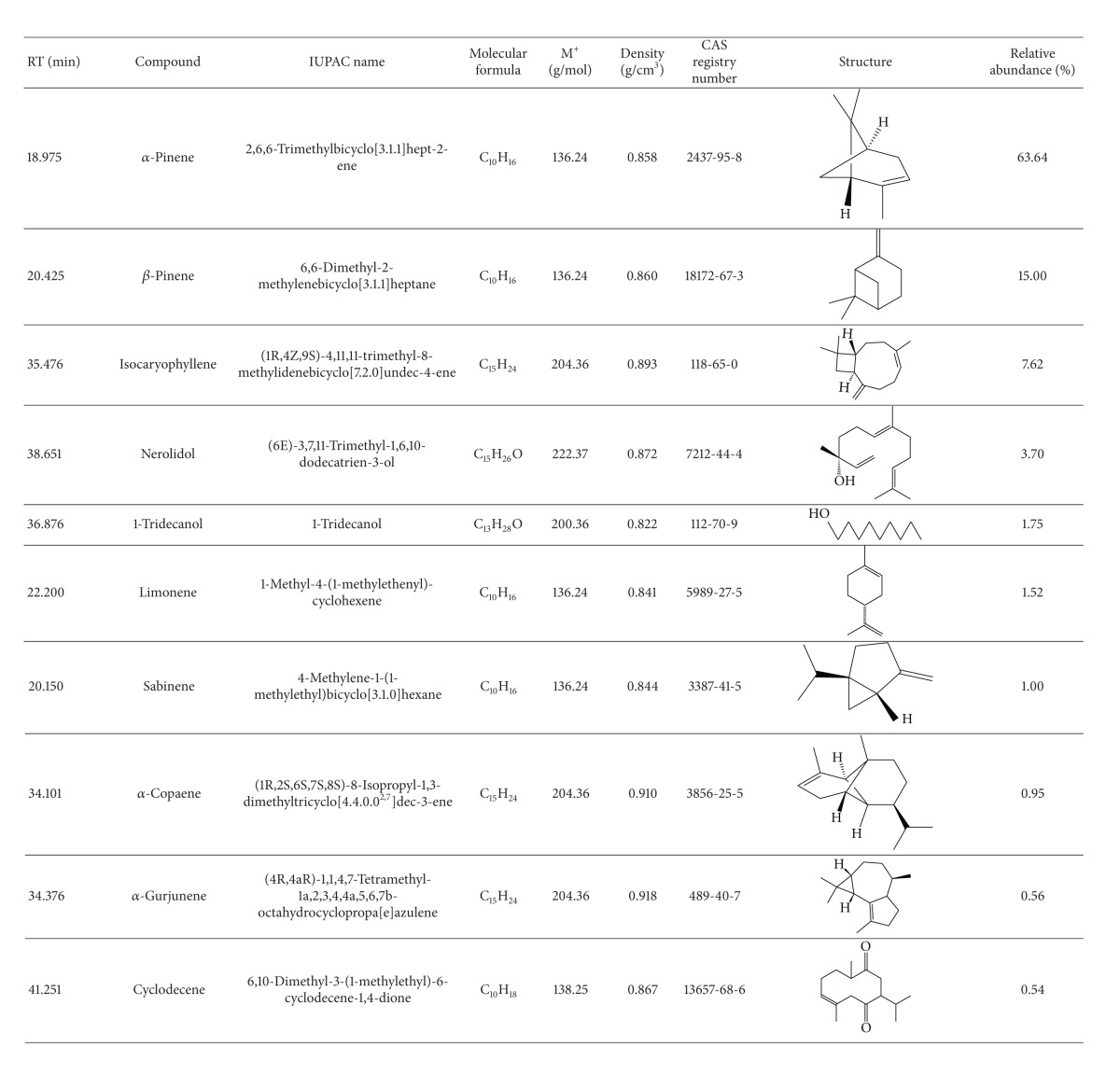

Key:

M^+^: molecular weight.

RT: retention time in minutes (min.).

IUPAC: The IUPAC nomenclature system of organic chemistry is a systematic method of naming organic chemical compounds as recommended by the International Union of Pure and Applied Chemistry (IUPAC).

CAS registry numbers: Are unique numerical identifier (values) numbers assigned by the chemical abstracts service (CAS) to every chemical described in the open scientific literature (currently including those chemicals described from at least 1957 through the present) and including elements, isotopes, organic and inorganic compounds, ions, organometallics, metals, nonstructurable materials, and so forth.

**Table 2:** Probit analysis of dose-response relationship of *Tagetes minuta* and *Tithonia diversifolia* essential oils at RD_50_ and RD_75_ generated by a regression model: Probit [Π(dose1)] = 1.1036 + 0.4132 dose1 for the essential oil of *T. minuta* and the regression model: Probit [Π(dose1)] = 0.6401 + 0.4962 dose1 for the essential oil of *T. diversifolia*.

Plant species	Repellence probability	Repellent dose (mg)	Upper confidence limit at 95%	Lower confidence limit at 95%
*Tagetes minuta *	0.50	0.0021	0.0024	0.0019
	0.75	0.0915	0.1012	0.0830
*Tithonia diversifolia *	0.50	0.2629	0.2712	0.2548
	0.75	0.5972	0.6116	0.5835

**Table 3:** Mean percent repellence (±SE) of N,N-diethyl-3-methylbenzamide (DEET) evaluated in a dual-choice assay against newly emerged adult ticks, *Rhipicephalus appendiculatus,* at the International Centre of Insect Physiology and Ecology, Nairobi, Kenya, under the same laboratory conditions as the current studies.

Repellent dose (mg)	Repellency (%)
0.0998	84.0 ± 3.9
0.00998	82.8 ± 3.6
0.000998	75.6 ± 4.5
0.0000998	70.5 ± 3.6

Sources: Ndung'u et al. [35], 1995; Lwande et al. [37], 1998; Ndung'u et al. [27], 1999.
